# Protein S-glutathionylation lowers superoxide/hydrogen peroxide release from skeletal muscle mitochondria through modification of complex I and inhibition of pyruvate uptake

**DOI:** 10.1371/journal.pone.0192801

**Published:** 2018-02-14

**Authors:** Robert M. Gill, Marisa O’Brien, Adrian Young, Danielle Gardiner, Ryan J. Mailloux

**Affiliations:** Memorial University of Newfoundland, Department of Biochemistry, St. John’s, Newfoundland, Canada; University of PECS Medical School, HUNGARY

## Abstract

Protein S-glutathionylation is a reversible redox modification that regulates mitochondrial metabolism and reactive oxygen species (ROS) production in liver and cardiac tissue. However, whether or not it controls ROS release from skeletal muscle mitochondria has not been explored. In the present study, we examined if chemically-induced protein S-glutathionylation could alter superoxide (O_2_^●-^)/hydrogen peroxide (H_2_O_2_) release from isolated muscle mitochondria. Disulfiram, a powerful chemical S-glutathionylation catalyst, was used to S-glutathionylate mitochondrial proteins and ascertain if it can alter ROS production. It was found that O_2_^●-^/H_2_O_2_ release rates from permeabilized muscle mitochondria decreased with increasing doses of disulfiram (100–500 μM). This effect was highest in mitochondria oxidizing succinate or palmitoyl-carnitine, where a ~80–90% decrease in the rate of ROS release was observed. Similar effects were detected in intact mitochondria respiring under state 4 conditions. Incubation of disulfiram-treated mitochondria with DTT (2 mM) restored ROS release confirming that these effects were associated with protein S-glutathionylation. Disulfiram treatment also inhibited phosphorylating and proton leak-dependent respiration. Radiolabelled substrate uptake experiments demonstrated that disulfiram inhibited pyruvate import but had no effect on carnitine uptake. Immunoblot analysis of complex I revealed that it contained several protein S-glutathionylation targets including NDUSF1, a subunit required for NADH oxidation. Taken together, these results demonstrate that O_2_^●-^/H_2_O_2_ release from muscle mitochondria can be altered by protein S-glutathionylation. We attribute these changes to the protein S-glutathionylation complex I and inhibition of mitochondrial pyruvate carrier.

## Introduction

Protein S-glutathionylation is a ubiquitous and reversible oxidative modification that controls protein function in response to changes in redox buffering capacity. In the cytosol, glutaredoxin-1 (GRX1) is the principle enzyme that mediates these reactions and is required to modulate a variety of cell programs ranging from gene expression to apoptosis [1,2]. Protein S-glutathionylation is also required to control various mitochondrial functions including oxidative phosphorylation and ROS release [3]. Complex I of the respiratory chain was identified as the first S-glutathionylation site in mitochondria [4,5]. Protein S-glutathionylation of complex I occurs at several sites including NDUFS1 and NDUFV1, subunits required for NADH oxidation and electron transfer to the ubiquinone binding site [5,6]. Complex I was also identified as the first site of action for GRX2, the matrix-bound isozyme for GRX1 [7]. GRX2 catalyzes the reversible S-glutathionylation of complex I in response to changes in glutathione buffering capacity, controlling its activity and the rate of ROS release [7,8]. Pyruvate dehydrogenase complex (PDHC) and α-ketoglutarate dehydrogenase complex (KGDHC), two important entry points for carbon into the Krebs cycle and sources of mitochondrial ROS, are also modulated by protein S-glutathionylation [9,10]. Evidence collected in several recent studies also indicates PDHC and KGDHC are targeted by GRX2 which lowers ROS release from both complexes following S-glutathionylation [9,11].

Protein S-glutathionylation is required to modulate several physiological functions ranging from heart contraction to eyesight [5,8]. Skeletal muscle contraction and metabolism have also been found to be modulated by protein S-glutathionylation [12]. For instance, ryanodine receptor-1 (RyR_1_), sarco/endoplasmic reticulum Ca^2+^ ATPase (SERCA), and Na^+^/K^+^ ATPase, transporters vital for muscle contraction and relaxation, are targets for protein S-glutathionylation [13,14]. In addition, various muscle contractile proteins, like troponin C and titin, are targets for S-glutathionylation [14,15]. Overall, redox signals conveyed through changes in antioxidant buffering capacity are required for muscle growth, fitness, and regulation of contraction/relaxation [16]. Skeletal muscle mitochondria are also important targets for regulation by protein S-glutathionylation. Most of the studies with muscle mitochondria have focused on understanding the relationship between S-glutathionylation and proton leaks. It was found that chemical induction of S-glutathionylation deactivates proton leaks in an uncoupling protein-3 (UCP3)-dependent manner [17]. Intriguingly, elimination of the *Grx2* gene maintained UCP3 in a deglutathionylated state increasing proton leaks and mitochondrial respiration [18].

ROS release from mitochondria has a bi-functional relationship with muscle tissue. Low-grade ROS release from mitochondria has been found to be beneficial for muscle growth and adaptation to exercise whereas over-production correlates with contractile dysfunction and the development of insulin resistance and obesity [19–21]. Thus, in order to benefit from the secondary signaling properties of ROS whilst avoiding oxidative distress, it is likely that skeletal muscle mitochondria invoke regulatory mechanisms to control how much O_2_^●-^/H_2_O_2_ is released. Here, we examined if the chemical induction of protein S-glutathionylation could alter O_2_^●-^/H_2_O_2_ release from isolated muscle mitochondria. Overall, our results demonstrate that protein S-glutathionylation can alter O_2_^●-^/H_2_O_2_ release from isolated muscle mitochondria through complex I modification or inhibition of the uptake of certain oxidizable substrates (*e*.*g*. pyruvate). The implications of these findings in understanding the regulation of mitochondrial ROS signaling in muscle are discussed further below.

## Experimental

### Chemicals

Diamide, disulfiram (Dis), NAD+, thiamine pyrophosphate (TPP), coenzyme A (CoASH), superoxide dismutase (SOD), horseradish peroxidase (HRP), HEPES, EGTA, Triton X-100, MgCl_2_, ATP, subtilisin A, Bradford reagent, pyruvate, 2-oxoglutarate, succinate, palmitoyl-carnitine, carnitine, ADP, oligomycin, antimycin A, malate and fatty acid free bovine serum albumin (BSA) were purchased from Sigma. Amplex Ultra Red (AUR) was purchased from Invitrogen. ^14^C-pyruvate and ^3^H-carnitine were purchased from PerkinElmer. Ecolume scintillation fluid was purchased from Fisher.

### Skeletal muscle mitochondria isolation

Memorial University’s Animal Care and Use Committee approved all animal experiments. Male C57Bl/6N mice were purchased from Charles River Laboratories at 8 weeks of age. At 10 weeks of age, mice were heavily anesthetized under isoflurane and euthanized by cerebral dislocation. All tissue extraction and mitochondrial isolation procedures were completed on ice or at 4°C. The forelimb, hindlimb, and pectoral muscles were excised and placed in basic medium (BM; 140 mM KCl, 20 mM HEPES, 5 mM MgCl_2_, 1 mM EGTA, pH 7.0). Connective tissue and fat were removed and the muscle tissue was dried and weighed. Samples were then minced on a Teflon plate and placed in a tissue homogenizer containing 30 mL of homogenizing medium (HM; BM containing 1 mM ATP, 1% BSA and 1 U subtilisin A). Tissue was homogenized using the Potter-Elvejham method. The homogenate was then centrifuged at 800 xg for 9 minutes. The supernatant was collected and centrifuged at 12000 xg for 9 minutes. The liquid was decanted and the pellet was resuspended in 1 mL of BM and incubated on ice for 5 minutes to promote the repolymerization of myofibers. Twenty-five milliliters of BM were then added to each tube and the samples centrifuged at 800 xg for 9 minutes. The supernatant was collected and centrifuged at 12000 xg for 9 minutes. The final mitochondria pellet was then resuspended in 200 μL of BM. Protein concentration was quantified using a Bradford assay using BSA as the standard. For experiments with permeabilized mitochondria, samples were diluted to 1.5 mg/mL in BM containing 0.5% v/v Triton X-100 and incubated on ice for 30 minutes [9].

### Amplex Ultra Red assay

O_2_^•-^/ H_2_O_2_ emission was measured using the AUR assay. To test the effect of protein S-glutathionylation on ROS release, permeabilized or intact mitochondria were first diluted in individual wells to a final concentration of 0.15 mg/mL and incubated in BM containing diamide (0 or 1000 μM) or disulfiram (0–500 μM), two documented chemical S-glutathionylation catalysts, at 25°C for 20 minutes [9,22,23]. SOD (25 U/mL), HRP (3 U/mL), and AUR (10 μM) were then added to each well followed by the addition of either pyruvate (50 μM), 2-oxoglutarate (50 μM), succinate (50 μM), or palmitoyl-carnitine (20 μM) to initiate the reaction. For experiments with intact mitochondria, malate (50 μM) was also added to reaction mixtures containing pyruvate or pre-loaded with carnitine (2 mM) for experiments with palmitoyl-carnitine. The final volume of each reaction was 200 μL. For experiments with permeabilized mitochondria, reaction mixtures were also supplemented with NAD+ (1 mM), TPP (0.3 mM), and CoASH (0.1 mM). In intact mitochondria oxidizing palmitoyl-carnitine, mitochondria were pre-incubated with carnitine (2 mM) for 5 minutes before exposure to diamide or disulfiram. O_2_^•-^/ H_2_O_2_ production was monitored using a spectra max plate reader (from Molecular Devices) and Softmax Pro software (version 5.4.6) which measured the H_2_O_2_ dependent conversion of AUR to fluorescent resorufin. Fluorescence was measured at an excitation: emission of 565:600 nm.

### Mitochondrial respiration

The different states of mitochondrial respiration were monitored using an Oxytherm electrode system (Hansatech). Mitochondria were incubated in disulfiram (0–500 μM) for 15 minutes and then diluted to 0.2 mg/ml in the reaction chamber containing BM supplemented with KH_2_PO_4_ (10 mM), MgCl_2_ (5 mM), and 0.1% w/v delipidated BSA. Mitochondria were allowed to equilibrate until a stable O_2_ baseline was reached and then reactions were initiated by the addition of either pyruvate (10 mM)/malate (2 mM), succinate (5mM) or palmitoyl carnitine (50 μM) to induce state 2 respiratory conditions. Note that for experiments with palmitoyl-carnitine, mitochondria were pre-incubated in the reaction chamber with carnitine (2 mM). After a few minutes, state 3 respiration (phosphorylating respiration) was stimulated by adding ADP (1 mM) followed by the induction of proton leak-dependent (state 4) respiration (oligomycin; 4 μg/ml). Oxygen consumption not associated with the respiratory chain was measured by adding antimycin A (4 μM). State 2–4 respiratory values were corrected for background O_2_ consumption (rates recorded following addition of antimycin A). Respiratory control ratio (RCR) values were calculated as the ratio of state 3 to state 4 respiration.

### TMRE assays

Membrane potential changes in mitochondria treated with or without disulfiram were tracked using TMRE under non-quench mode conditions. Mitochondria were diluted to 0.15 mg/mL in BM supplemented with KH_2_PO_4_ (10 mM), MgCl_2_ (5 mM), and 0.1% w/v delipidated BSA in 96-welled black plates and then treated with or without disulfiram (0–500 μM) and incubated for 15 minutes at 25°C. Samples were then supplemented with pyruvate (50 μM) and malate (50 μM) and succinate (50 μM) to stimulate state 4 respiration. For some reactions, ADP (1 mM) was also added to stimulate state 3 respiration. Following the addition of the different substrates, TMRE was added to each well (10 nM) and fluorescent changes in membrane potential were tracked at ex:em of 549 nm:575 nm.

### Solute import assays

The uptake of pyruvate or carnitine by mitochondria was measured as described in [24]. Mitochondria were diluted to 0.2 mg/mL in BM containing succinate to polarize the mitochondrial inner membrane and then incubated in disulfiram (0μM, 100 μM, 200 μM or 500 μM) for 10 minutes at 25°C. Reaction mixtures were then supplemented with ^3^H-carnitine or ^14^C-pyruvate (100 μM final concentration) with 100 μM cold pyruvate or carnitine and incubated for 10 minutes at 25°C. Samples were then centrifuged at 10,000 xg for 10 minutes and the mitochondrial pellet was then washed thrice with ice-cold BM. The final mitochondrial pellet was resuspended in 100 μL BM and placed in scintillation vials containing 5 mL of Ecolume cocktail. Samples were allowed to settle for counted for 5 minutes on a liquid scintillation counter (Tri Carb 2810).

### Protein S-glutathionylation adducts

Mitochondria were diluted to 3 mg/mL in BM containing 0 μM or 1000 μM diamide and pyruvate and malate (50 μM), 2-oxoglutarate (50 μM), succinate (50 μM), or palmitoyl-carnitine (50 μM) and incubated for 20 min at 25°C. Samples were diluted to 1.5 mg/mL in Laemmli buffer and heated for 10 min at 100°C. Samples were then electrophoresed under nonreducing conditions in a 10% isocratic acrylamide denaturing gel. Separated proteins were then electroblotted to nitrocellulose membranes. Successful transfer was confirmed by staining membranes with Ponceau S. Membranes were then cut laterally just above the 28 KDa molecular weight marker with a razor. The top part of the membrane was used to probe for protein glutathione mixed disulfide (PSSG) adducts and the bottom used as the loading control. Membranes were blocked for 1 hour at room temperature under constant agitation with tris-buffered saline (TBS) containing 0.1% (v/v) tween-20 (TBS-T) and 5% (w/v) non-fat skim milk (blocking solution). Membranes were then washed twice with TBS-T and probed overnight at 4°C under constant agitation with protein glutathione mixed disulfide (PSSG; 1/500 dilution, Santa Cruz) antiserum or MnSOD (loading control. 1/3000, Santa Cruz), diluted in TBS-T containing 5% (w/v) BSA and 0.02% (w/v) NaN_3_. Membranes were then washed twice with TBS-T and probed with goat anti-mouse (1/2000, Abcam) or anti-rabbit horseradish peroxidase conjugate (1/2000, Santa Cruz) diluted in blocking solution for 1 hour at room temperature. Bands were visualized using WestPico Super Signal Chemiluminescent substrate and ImageQuant LAS 4000.

### Complex I immunocapture

Complex I was immunopurified using an immunocapture kit purchased from Mitosciences. Mitochondria were diluted to 5 mg/mL in phosphate buffered saline (PBS) containing 500 μM disulfiram in a final volume of one hundred microliters. Reaction mixtures devoid of disulfiram served as the control. Mitochondrial suspensions were then vortexed vigorously and incubated at 25°C for 20 minutes, mixing the reactions at 5-minute intervals. Complex I was then immunopurified according to the manufacturer’s instructions (Mitosciences). Briefly, mitochondria were solubilized with 10 μL of extraction buffer and then incubated overnight in 10 μL of bead slurry at 4°C under constant gentle agitation. Beads were then washed three times with PBS and complex I was eluted using SDS elution buffer according to the manufacturer’s instructions. The supernatant was then collected and stored at -80°C.

Samples were diluted in Laemmli Buffer and then heated at 100°C for 10 min. Note that samples were electrophoresed under nonreducing conditions unless specified otherwise—reducing electrophoresis required supplementing Laemmli buffer with 2% (v/v) β-mercaptoethanol, a reducing agent that reverses protein S-glutathionylation. Protein samples were then electrophoresed through a 10% isocratic denaturing acrylamide gel and then transferred to nitrocellulose membranes for immunoblot. Successful transfer of proteins was monitored by staining membranes with Ponceau S solution. Membranes were then quickly washed with Tris-buffered saline (TBS) containing 0.1% (v/v) tween-20 (TBS-T) and then blocked for 1 hour under constant agitation with TBS-T containing 5% (w/v) non-fat skim milk. Membranes were then washed three times with TBS-T and probed with anti-OXPHOS antibody cocktail (1/3000, Abcam), anti-NDUFS1 (1/3000, Abcam), or anti-protein glutathione mixed disulfide (PSSG; 1/500, Santa Cruz) diluted in TBS-T containing 5% (w/v) defatted BSA overnight at 4°C under constant agitation. Membranes were then washed three times with TBS-T and probed with goat anti-mouse horseradish peroxidase conjugate diluted in blocking solution (1/3000, Abcam). Bands were visualized as described above.

### Data analysis

Amplex Ultra Red assays were performed 4 times and in duplicate. Polarographic measure of mitochondrial respiration was conducted 4 times and in duplicate. Immunocapture assays were conducted in triplicate. All results were analyzed using GraphPad Prism 6 software using a paired two-tailed Student T-Test or a 1-way ANOVA with a Tukey’s posthoc test. *; p ≤ 0.05, ** and ##; p ≤ 0.01, ***; p ≤ 0.001. ****; P ≤ 0.0001.

## Results

### Disulfiram lowers ROS release from muscle mitochondria

**Fig 1:**
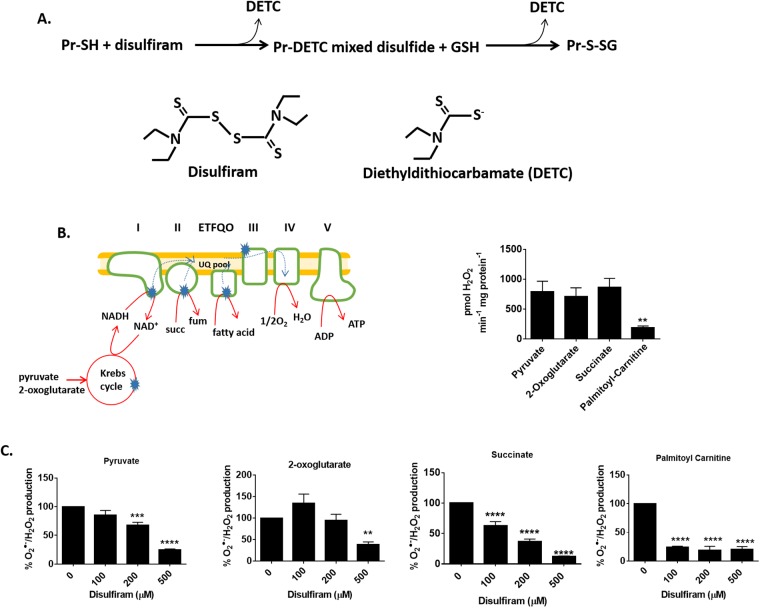
S-glutathionylation catalyst disulfiram inhibits ROS release from permeabilized skeletal muscle mitochondria. (A) A diagram depicting the chemical induction of protein S-glutathionylation by disulfiram. (B) Depiction of the entry point for electrons into the respiratory chain in mitochondria oxidizing different substrates. Dotted lines correspond to the direction of electron flow. Note that electrons from complex II and ETFQO (electron transfer flavoprotein-ubiquinone oxidoreductase) can flow backwards to complex I under state 4 respiratory conditions. Blue stars indicate sites for ROS release. O_2_^●-^/H_2_O_2_ release by permeabilized muscle mitochondria oxidizing 50 μM pyruvate, 2-oxoglutarate, succinate, or palmitoyl-carnitine was tracked for 5 minutes using Amplex UltraRed. n = 4, mean±SEM. (C) Permeabilized skeletal muscle mitochondria were treated for 20 min with disulfiram (100–500 μM) and then O_2_^●-^/H_2_O_2_ release was tracked using Amplex UltraRed. The final concentration of pyruvate, 2-oxoglutarate, succinate, or palmitoyl-carnitine was 50 μM. n = 4, mean±SEM.

Disulfiram contains reactive thiol groups that have been shown to induce the rapid and highly specific S-glutathionylation of protein cysteine thiols [25]. It was demonstrated in a recent study that disulfiram is approximately twenty times more effective than diamide at inducing the S-glutathionylation of mitochondrial proteins [9]. In addition, disulfiram is highly effective at limiting ROS release from PDHC and KGDHC, two potent sources of O_2_^●-^/H_2_O_2_ in liver mitochondria [9]. Therefore, we used disulfiram to assess the effect of S-glutathionylation on ROS release from skeletal mitochondria. The mechanism for disulfiram-induced protein S-glutathionylation is likely similar to the diamide-mediated conjugation of glutathione to a cysteine residue [26]. Disulfiram contains thiols which may react with a protein cysteine residue forming a mixed disulfide (Fig 1A) [25]. Reduced glutathione then displaces diethyldithiocarbamate (DETC) producing an S-glutathionylated protein.

The capacity of disulfiram to limit the rate of O_2_^●-^/H_2_O_2_ release was tested using different carbon substrates that feed electrons into different parts of the electron transport chain (ETC) (Fig 1B). Pyruvate and 2-oxoglutarate are oxidized by PDHC and KGDHC, respectively, and the resulting NADH is oxidized by complex I. Succinate and palmitoyl-carnitine donate electrons directly to the ubiquinone pool in the ETC via oxidation by complex II and electron-transfer flavoprotein:ubiquinone oxidoreductase (ETFQO), respectively. Notably, PDHC, KGDHC, complex II have been documented to be sites for high ROS release in muscle mitochondria whereas ETFQO is not a significant source [27]. Pyruvate, 2-oxoglutarate, and succinate, at a final concentration of 50 μM, induced a robust rate of O_2_^●-^/H_2_O_2_ production by permeabilized muscle mitochondria (Fig 1B). Palmitoyl-carnitine also induced O_2_^●-^/H_2_O_2_ production but the rate of ROS release was ~4-fold lower when compared to pyruvate, 2-oxoglutarate, or succinate. Next, we examined if disulfiram could impede ROS release from permeabilized mitochondria. It was found that at least 200 μM disulfiram was required to significantly decrease ROS release during pyruvate or 2-oxoglutarate oxidation (Fig 1C). In addition, 500 μM induced ~80% decrease in ROS release when pyruvate and 2-oxoglutarate was being metabolized. Disulfiram was more effective at limiting ROS release from permeabilized mitochondria when succinate and palmitoyl-carnitine served as substrates. Indeed, 100 μM disulfiram was sufficient to induce a significant decrease in ROS release during succinate oxidation (Fig 1C). In addition, maximal inhibition of O_2_^●-^/H_2_O_2_ production during succinate metabolism was obtained when mitochondria were incubated in 500 μM disulfiram (~90% decrease in release). On the other hand, 100 μM disulfiram decreased ROS release by ~90% when palmitoyl-carnitine served as the substrate (Fig 1C).

**Fig 2:**
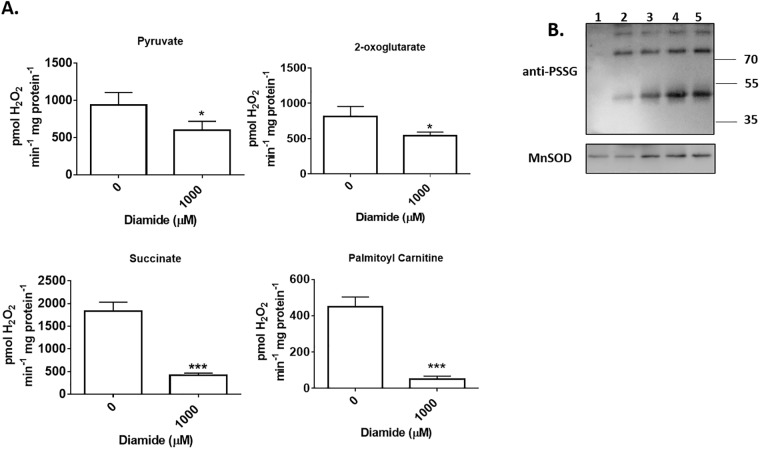
S-glutathionylation catalyst diamide also decreases ROS release from permeabilized skeletal muscle mitochondria. (A) Mitochondria were incubated in diamide (1000 μM) for 20 min and then O_2_^●-^/H_2_O_2_ release was tracked using Amplex UltraRed. The final concentration of pyruvate, 2-oxoglutarate, succinate, or palmitoyl-carnitine was 50 μM. n = 4, mean±SEM. (B) The decrease in mitochondrial ROS release correlates with an increase in overall protein S-glutathionylation. Protein S-glutathionylation was induced with 1000 μM diamide. PSSG levels were assessed in mitochondria incubated in; pyruvate and malate alone (lane 1), pyruvate and malate with diamide (lane 2), 2-oxoglutarate with diamide (lane 3), succinate with diamide (lane 4), and palmitoyl-carnitine with diamide (lane 5). Changes in PSSG levels were assessed by immunoblot. MnSOD served as the loading control.

The above results indicate that disulfiram is more effective at limiting O_2_^●-^/H_2_O_2_ production when the oxidizable carbon source bypasses the Krebs cycle and donates electrons directly to the respiratory chain. We decided to confirm these findings using diamide, a commonly used catalyst for S-glutathionylation. Diamide inhibited ROS release from permeabilized mitochondria oxidizing pyruvate and 2-oxoglutarate but only by ~40% (Fig 2A). Diamide also impeded O_2_^●-^/H_2_O_2_ production in mitochondria oxidizing succinate and palmitoyl-carnitine but by ~90% (Fig 2A). Assessment of the overall number of protein glutathione mixed disulfides revealed that the diamide induced decrease in ROS release correlated with increased mitochondrial protein S-glutathionylation (Fig 2B). Overall, these results demonstrate that induction of protein S-glutathionylation can decrease ROS release from permeabilized mitochondria.

### Disulfiram also inhibits ROS release from intact muscle mitochondria

**Fig 3:**
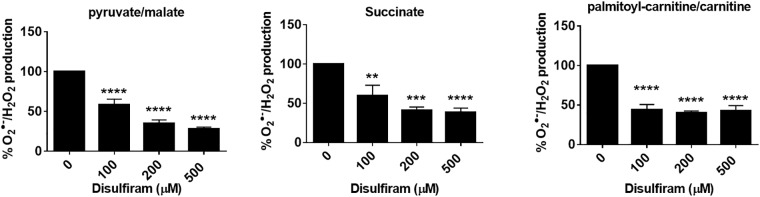
Disulfiram decreases ROS release from skeletal muscle mitochondria with an intact mitochondrial inner membrane. Mitochondria were treated with disulfiram (100–500 μM) for 20 min and then O_2_^●-^/H_2_O_2_ release was tracked using Amplex UltraRed. The final concentration of pyruvate, succinate, or palmitoyl-carnitine was 50 μM. For experiments with pyruvate, malate was also included in the reaction mixture at a final concentration of 50 μM. In palmitoyl-carnitine expeirments, mitochondria were pre-incubated in basic medium containing 2 mM carnitine.

Use of permeabilized mitochondria to study ROS release has the advantage of eliminating the proton gradient, a factor that can interfere with quantifying O_2_^●-^/H_2_O_2_ production rates from different sites of formation. Nonetheless, under physiological conditions, membrane potential strength can influence ROS release from mitochondria *in vivo*. Therefore, we examined the effect of ROS release from intact mitochondria operating under state 4 respiratory conditions. Under these conditions, disulfiram had a similar effect on ROS release in mitochondria oxidizing pyruvate, succinate, or palmitoyl-carnitine (Fig 3A). In mitochondria oxidizing pyruvate, 100 μM disulfiram decreased ROS release by almost 50%. Higher disulfiram concentrations (200 μM and 500 μM) had a stronger effect on O_2_^●-^/H_2_O_2_ production, inhibiting ROS release by ~75% (Fig 3A). Similar results were collected with both succinate and palmitoyl-carnitine. Indeed, 100 μM disulfiram was sufficient to inhibit ROS release by ~50% in mitochondria oxidizing succinate or palmitoyl-carnitine (Fig 3A).

**Fig 4:**
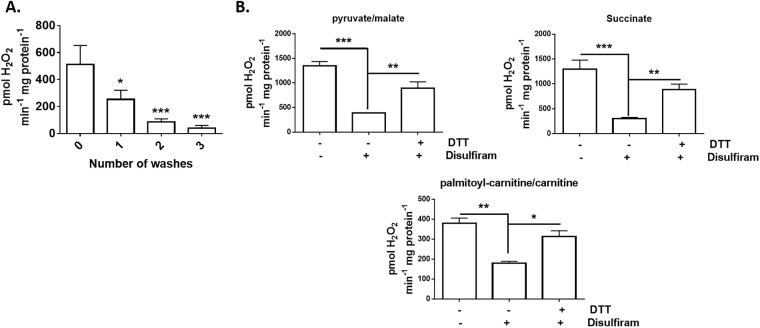
Deglutathionylation agent, DTT, restores ROS release from skeletal muscle mitochondria treated with disulfiram. (A) Auto-stimulation of O_2_^●-^/H_2_O_2_ release by DTT can be prevented by washing mitochondria. For experiments, mitochondria were not supplemented with substrate. Mitochondria were treated with DTT, incubated for 10 min, and then washed 0–3 times with basic medium. Spontaneous O_2_^●-^/H_2_O_2_ release was then recorded. (B) Mitochondria were treated with disulfiram (500 μM) for 20 min, washed once, and then treated with or without DTT (2 mM) for an additional 10 min. Mitochondria were then washed thrice and O_2_^●-^/H_2_O_2_ release was tracked using Amplex UltraRed. The final concentration of pyruvate, succinate, or palmitoyl-carnitine was 50 μM. For experiments with pyruvate, malate was also included in the reaction mixture at a final concentration of 50 μM. In palmitoyl-carnitine experiments, mitochondria were pre-incubated in basic medium containing 2 mM carnitine. n = 4, mean±SEM.

Next, we decided to test if the disulfiram effect could be reversed with a reducing agent. Dithiothreitol (DTT) is a documented to deglutathionylation agent but it has been recently shown that it can stimulate high rates of ROS release by PDHC or KGDHC or intact mitochondria in the absence of substrate [28]. Since we were interested in using DTT to ascertain if we could reverse the disulfiram-mediated suppression of ROS release, we first tested if washing mitochondria after a DTT treatment could prevent it from auto-stimulating mitochondrial O_2_^●-^/H_2_O_2_ production. Fig 4A demonstrates that DTT was able to induce a robust increase in mitochondrial ROS production in the absence of substrate. However, successive washes significantly reduced DTT-stimulated mitochondrial O_2_^●-^/H_2_O_2_ formation. Subjecting mitochondria to one wash with basic medium lowered ROS release by ~50% (Fig 4A). Washing mitochondria, a second time, decreased resorufin fluorescence by ~84%. Subjecting mitochondria to a third wash almost completely abolished ROS release (decreased by ~93%) (Fig 4A). Mitochondria treated with 500 μM disulfiram displayed a significant decrease in ROS release, regardless of substrate. However, incubation of disulfiram-treated mitochondria with DTT (2 mM) followed by three successive washes reversed this effect, recovering the rate of O_2_^●-^/H_2_O_2_ production (Fig 4B). Moreover, DTT was able to recover ROS release in mitochondria oxidizing pyruvate, succinate, or palimtoyl-carnitine.

### Disulfiram decreases mitochondrial respiration

**Fig 5:**
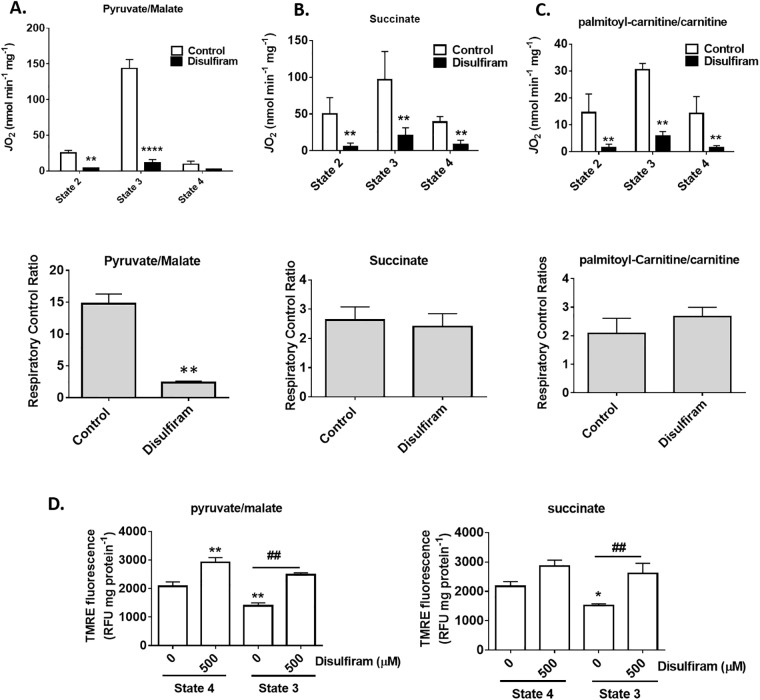
Disulfiram impedes mitochondrial respiration. Mitochondria were pre-incubated with disulfiram (100 μM) and the different states of respiration were measured. State 2 respiration was induced by the addition of pyruvate/malate (A), succinate (B), or palmitoyl-carnitine (C). The final concentration of pyruvate/malate, succinate, or palmitoyl-carnitine was 10 mM/2 mM, 5 mM, and 50 μM, respectively. State 3 (phosphorylating respiration) and state 4 (proton leak-dependent respiration) were induced by addition ADP (1 mM) and oligomycin (4 μg/mL) to the chamber. Antimycin A was then added to assess O_2_ consumption not associated with the respiratory chain. All respiration values were corrected for O_2_ consumption not associated with the electron transport chain. (D) Membrane potential determinations were conducted on mitochondria treated with disulfiram (100–500 μM) and TMRE (10 nM). The final concentration of pyruvate, succinate, or palmitoyl-carnitine was 50 μM. For experiments with pyruvate, malate was also included in the reaction mixture at a final concentration of 50 μM. In palmitoyl-carnitine expeirments, mitochondria were pre-incubated in basic medium containing 2 mM carnitine. * corresponds to significance when compared to state 4 control. # corresponds to significance when comparing control to disulfiram during state 3 or 4 respiration. n = 4, mean±SEM.

The results collected above indicate that disulfiram alters mitochondrial ROS release through S-glutathionylation. ROS release from mitochondria is highly dependent on the entry and exit of electrons from enzymes and respiratory complexes involved in respiration and ATP production. Therefore, it is possible that the disulfiram-induced decrease in ROS release is associated with a decrease in electron flow and delivery in the respiratory chain. To test this hypothesis, we examined the different states of respiration in mitochondria oxidizing pyruvate, succinate, or palmitoyl-carnitine. Supplementing mitochondria oxidizing pyruvate with ADP induced a robust increase in state 3 (phosphorylating) respiration indicating that membranes were intact and well coupled (Fig 5A). Treatment of mitochondria with 500 μM disulfiram induced a significant decrease in state 3 respiration. The almost complete abolishment of state 3 respiration by disulfiram induced a significant decrease in the respiratory control ratio (RCR), a proxy measure for ATP producing capacity (Fig 5A). Next, we tested if disulfiram would have a similar effect on mitochondria oxidizing succinate. Disulfiram induced significant decreases in proton leak dependent respiration (state 2 and state 4) and phosphorylating respiration (Fig 5B). Despite this negative effect, no differences in RCR were observed, an effect attributed to the increase in leaks associated with succinate metabolism. Similar observations were made with palmitoyl-carnitine. Disulfiram (500 μM) induced a sharp decrease in leak and phosphorylating respiration (Fig 5C). However, no effect on ATP forming potential was observed between control and disulfiram-treated mitochondria (Fig 5C).

Next, we examined if the disulfiram induced changes in mitochondrial respiration corresponded to alterations in membrane potential. Mitochondria treated with disulfiram displayed a significant increase in membrane potential when pyruvate and malate served as substrates (Fig 5D). Stimulation of state 3 respiration resulted in a significant decrease in TMRE fluorescence (Fig 5D). Intriguingly, the induction of state 3 respiration in mitochondria treated with disulfiram did not result in any significant change in TMRE fluorescence. We confirmed these observations using succinate as a substrate (Fig 5D). It was found that disulfiram increased TMRE fluorescence in mitochondria oxidizing succinate. Inclusion of ADP resulted in a decrease in TMRE fluorescence in control mitochondria (Fig 5D). However, the addition of disulfiram inhibited the ADP-induced decrease in TMRE fluorescence. Overall, these results confirm our observation that disulfiram inhibits phosphorylating respiration.

### Disulfiram induces the S-glutathionylation of complex I

**Fig 6:**
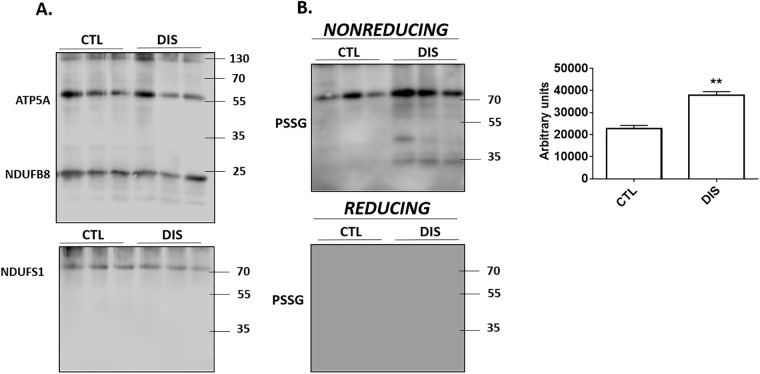
Disulfiram S-glutathionylates several complex I subunits. Mitochondria treated with or without 500 μM disulfiram and then complex I was immunopurified. (A) Immunodetection of the presence of subunits corresponding to complexes I-V with OXPHOS antibody cocktail and NDUFS1 subunit for complex I. n = 3. (B) Immunodetection of PSSG adducts in immunopurified complex I. Samples were also electrophoresed under reducing conditions to confirm PSSG anti-serum specificity. Degree of overall protein S-glutathionylation for samples treated with or without disulfiram was quantified using Image J software. n = 4, mean±SEM.

Next, we tested if disulfiram could S-glutathionylate complex I. Mitochondria were incubated in 500 μM disulfiram and then complex I was immunocaptured for western blot analysis. Fig 6A demonstrates that we successfully immunopurified complex I. Strong immunoreactive bands corresponding to complex I subunit NDUFB8 were detected in all samples probed with the OXPHOS antibody cocktail. The OXPHOS cocktail contains antibodies directed against select subunits for the five respiratory complexes. Subunits corresponding to complexes II-IV were not detected (Fig 6A). However, we did detect the ATP5A subunit for complex V. This would indicate that our immunoprecipitation isolated both complex I and complex V. It needs to be emphasized though that complex I does form supercomplexes with complex V and that it is one of the most abundant proteins in muscle mitochondria. To confirm the successful enrichment of complex I, we probed for NDUFS1 (Fig 6A). We only detected one immunoreactive band which corresponded to the molecular weight of NDUFS1 confirming the successful enrichment of complex I. Next, we probed our samples for the presence of protein-glutathione disulfide adducts (PSSG). As shown in Fig 6B, treatment with disulfiram led to a significant increase in the intensity of a band corresponding to the molecular weight of NDUFS1. In addition, several other immunoreactive bands were detected (Fig 6B). Overall, disulfiram treatment induced a significant increase in the total number of PSSG adducts. Samples were also electrophoresed with β-mercaptoethanol, a reducing agent that reverses PSSG adduct formation. Inclusion of β-mercaptoethanol abolished all immunoreactive bands indicating that the bands detected under nonreducing electrophoresis conditions corresponded to PSSG adducts (Fig 6B).

### Effect of disulfiram on pyruvate and carnitine uptake

**Fig 7:**
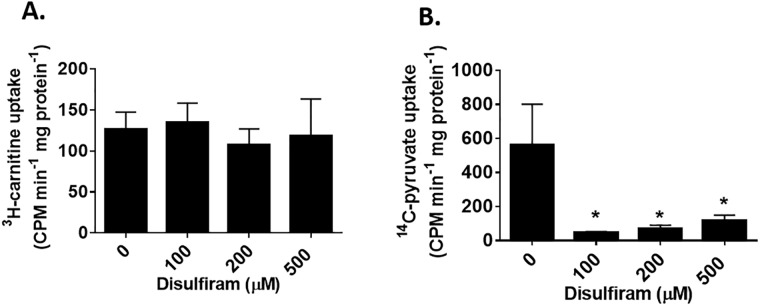
Disulfiram inhibits pyruvate import but not carnitine uptake. (A) Mitochondria were incubated in ^3^H-carnitine (100 μM) and cold carnitine (100 μM) for 10 minutes. Mitochondria were then washed twice and the amount of tritiated-carnitine take up was measured. (B) Mitochondria were incubated in ^14^C-pyruvate (100 μM) and cold pyruvate (100 μM) for 10 minutes. Mitochondria were then washed twice and the amount of tritiated-carnitine take up was measured. n = 4, mean±SEM.

Disulfiram is not a site specific chemical catalyst for the S-glutathionylation of proteins. Solute anion carrier proteins in mitochondria have been shown to undergo S-glutathionylation which can inhibit transport activity [24]. Therefore, we reasoned that the inhibition of mitochondrial ROS release by disulfiram could be associated with the deactivation of substrate uptake. We chose to examine uptake by the carnitine/acyl-carnitine character (CAC) and pyruvate carrier since either substrate drives ROS release from different enzymes. As shown in Fig 7A, it was found that disulfiram did not alter the uptake of ^3^H-carnitine. Indeed, even at 500 μM disulfiram, ^3^H-carnitine uptake was not changed with respect to control mitochondria. Next, the uptake of ^14^C-pyruvate was tested. Intriguingly, we observed that 100 μM disulfiram induced a robust and significant decrease in ^14^C-pyruvate uptake (~91% inhibition) (Fig 7B). In addition, similar observations were made at higher concentrations of disulfiram (Fig 7B). These results indicate that disulfiram inhibits palmitoyl-carnitine driven ROS release by S-glutathionylation mitochondrial enzymes like complex I. Inhibition of ROS release during pyruvate oxidation on the other hand is related to the deactivation of mitochondrial pyruvate carrier.

## Discussion

Protein S-glutathionylation reactions are integral for modulating mitochondrial function in response to changes in overall redox buffering capacity. Changes in the availability of reduced and oxidized glutathione serves a “*rheostat*” for mitochondria, S-glutathionylating proteins in response to changes in H_2_O_2_ and NADPH availability to control various functions [29,30]. Protein S-glutathionylation occurs in response to increased ROS and glutathione pool oxidation which is subsequently reversed upon restoration of matrix GSH levels [31]. Modification of proteins by protein S-glutathionylation in response to high H_2_O_2_ has been shown to protect enzymes like KGDHC and complex I from irreversible oxidative deactivation [6,32]. Restoration of mitochondrial GSH levels and a decrease in overall ROS levels results in protein deglutathionylation and enzyme reactivation. The S-glutathionylation of proteins in mitochondria has also been found to serve as a negative feedback loop for controlling ROS release. For instance, S-glutathionylation suppresses ROS release from KGDHC and PDHC, significant sources of O_2_^●-^/H_2_O_2_ in liver mitochondria [9]. The oxidative modification of complex I and II with glutathione also limits ROS release from either enzyme complex in cardiac mitochondria [6,33]. Collectively, protein S-glutathionylation serves as an important ROS release regulator.

The present study reveals that ROS release from isolated skeletal muscle mitochondria can be altered by chemically induced protein S-glutathionylation. It was found that disulfiram limited ROS release from permeabilized mitochondria during the oxidation of Krebs cycle linked substrates (pyruvate and 2-oxoglutarate) and carbon sources that donate electrons directly to the ubiquinone pool (succinate and palmitoyl-carnitine). These findings are consistent with a previous study where it was shown that diamide and disulfiram induced a significant decrease in ROS release in permeabilized liver mitochondria oxidizing pyrurvate or 2-oxoglutarate [9]. However, to our knowledge this is the first time that the impact of protein S-glutathionylation on palmitoyl-carnitine driven ROS production and respiration has ever been examined *in vitro*. Here, it was also found that complex I is a major site for disulfiram-induced S-glutathionylation. Complex I can be subjected to reversible S-glutathionylation in response to changes in reduced glutathione availability, a reaction catalyzed by GRX2 [7]. S-glutathionylation targets include NDUFS1, NDUFV1, and ND3, subunits required for complex I activity [34]. Transient S-glutathionylation of NDUFS1 does decrease ROS release from mitochondria [6]. This effect was postulated to be associated with blockage of electron flow and inhibition of the reduction of FMN [6]. Here, we observed that complex I has several S-glutathionylation targets in skeletal muscle mitochondria including NDUFS1. It needs to be acknowledged that rat skeletal muscle mitochondria contain a number of important ROS release sites. KGDHC and PDHC can form ~8x and ~4x more ROS than complex I during the oxidation of Krebs cycle metabolites and complexes II and III also serve as important sites for O_2_^●-^/H_2_O_2_ release [27]. By contrast, complexes I and III and to a lesser extent II are high capacity sites during the oxidation of carbon sources that donate electrons directly to the ubiquinone pool [27]. Therefore, we cannot discount that disulfiram may be inhibiting ROS release from other producers in permeabilized muscle mitochondria, like PDHC or KGDHC. However, our results do indicate that inhibition of O_2_^●-^/H_2_O_2_ release from muscle mitochondria occurs, in part, due to the S-glutathionylation of complex I.

A previous study showed that CAC can be S-glutathionylated on Cys^136^ and Cys^155^ and that this modification was highly sensitive to the availability of reduced glutathione [24]. Moreover, the same study found that S-glutathionylation of recombinant CAC can be mediated by purified GRX1 [24]. Here, it was found that disulfiram did not inhibit carnitine uptake. This would indicate that inhibition of ROS release following treatment of intact mitochondria with disulfiram is associated with the S-glutathionylation of complex I and perhaps other O_2_^●-^/H_2_O_2_ emitting sites. Indeed, complex I has been found to be the main source of ROS release during palmitoyl-carnitine oxidation [35]. Reverse electron flow from the ubiquinone pool to complex II and forward transfer to complex III were also found to be important ROS release sites from muscle mitochondria oxidizing palmitate [35]. We made similar observations with succinate, which forms ROS by reverse electron flow to complex I when the membrane potential is high [36]. It should be noted that the S-glutathionylation of CAC was originally studied in recombinant protein reconstituted in liposomes [24]. By contrast, we assessed carnitine import by CAC in isolated skeletal muscle mitochondria treated with disulfiram, a highly effective S-glutathionylation agent. Overall, our results demonstrate that disulfiram-induced S-glutathionylation limits ROS release by blocking electron flow in the respiratory chain, potentially through the S-glutathionylation of complex I, a major source of O_2_^●-^/H_2_O_2_ during reverse electron flow from the ubiquinone pool.

In the present study, we made the observation that disulfiram strongly inhibited pyruvate import by intact muscle mitochondria. To our knowledge this is the first time it has been reported that pyruvate uptake may be governed by a redox switch. Therefore, inhibition of ROS release in intact mitochondria oxidizing pyruvate may be partly associated with the deactivation of mitochondrial pyruvate carrier. Several studies have found that members of the solute anion carrier superfamily can undergo protein S-glutathionylation. For instance, ADP/ATP transporter, also referred to as adenine nucleotide translocator (ANT), can be S-glutathionylated, which was shown to prevent mitochondrial permeability transition pore opening [37]. Other documented targets include members of the UCP protein family. S-glutathionylation of UCP2, which is more ubiquitously expressed, was found to regulate insulin release by altering mitochondrial ROS release in pancreatic β-cells and sensitize drug resistant promyelocytic leukemia cells to chemotherapy [38,39]. Likewise, S-glutathionylation of UCP3 has been shown to regulate mitochondrial energy metabolism and inhibit proton leaks [17]. We made a similar observation in this study where it was found that disulfiram induced a significant decrease in proton leaks, particularly in mitochondria oxidizing succinate or palmitoyl-carnitine. UCP3 is S-glutathionylated on Cys^259^, which is found on the last loop region of the protein that makes contact with the matrix [17]. Recent work has shown that UCP1 in brown adipose tissue is also modulated by a cysteine switch (Cys^253^) located on its last loop region in the matrix [40]. Moreover, this cysteine on UCP1-3 is surrounded by basic amino acids making it more amenable for oxidative modification. Other solute anion carrier proteins, like ANT, also harbor an accessible cysteine in its last matrix loop region [41]. Intriguingly, mitochondrial pyruvate carrier also has a cysteine located on its last loop region surrounded by basic amino acids [42]. Although speculative at this point, this cysteine may represent the site for regulation of the mitochondrial pyruvate carrier by S-glutathionylation.

## Conclusions

It has been documented that protein S-glutathionylation modulates a number of mitochondrial functions including nutrient metabolism and ROS release [43]. Studies have found that this redox-sensitive modification can control O_2_^●-^/H_2_O_2_ production by PDHC, KGDHC, complex I and complex II in response to changes in H_2_O_2_ and NADPH availability in liver, lens, and heart mitochondria [6,9,33]. Here, we made similar observations with muscle mitochondria. The observation that protein S-glutathionylation lowers ROS release by mitochondria, in part by modifying complex I and decreasing solute uptake, indicates that it may be integral for controlling O_2_^●-^/H_2_O_2_ signaling in muscle. Indeed, mitochondrial O_2_^●-^/H_2_O_2_ emission is required for muscle adaptation and growth following exercise. Protein S-glutathionylation may be required to desensitize the ROS signal following muscle contraction. Ongoing studies by our group are currently invested in deciphering if mitochondrial protein S-glutathionylation reactions are required for adaptation to exercise through control of ROS production.

## References

[1] Allen EM, Mieyal JJ (2012) Protein-thiol oxidation and cell death: regulatory role of glutaredoxins. Antioxid Redox Signal 17: 1748–1763. doi: 10.1089/ars.2012.4644 22530666 10.1089/ars.2012.4644 PMC3474186

[2] Cooper AJ, Pinto JT, Callery PS (2011) Reversible and irreversible protein glutathionylation: biological and clinical aspects. Expert Opin Drug Metab Toxicol 7: 891–910. doi: 10.1517/17425255.2011.577738 21557709 10.1517/17425255.2011.577738 PMC3116085

[3] Mailloux RJ, Jin X, Willmore WG (2014) Redox regulation of mitochondrial function with emphasis on cysteine oxidation reactions. Redox Biol 2: 123–139. doi: 10.1016/j.redox.2013.12.011 24455476 10.1016/j.redox.2013.12.011 PMC3895620

[4] Taylor ER, Hurrell F, Shannon RJ, Lin TK, Hirst J, Murphy MP (2003) Reversible glutathionylation of complex I increases mitochondrial superoxide formation. J Biol Chem 278: 19603–19610. doi: 10.1074/jbc.M209359200 12649289 10.1074/jbc.M209359200

[5] Wu H, Lin L, Giblin F, Ho YS, Lou MF (2011) Glutaredoxin 2 knockout increases sensitivity to oxidative stress in mouse lens epithelial cells. Free Radic Biol Med 51: 2108–2117. doi: 10.1016/j.freeradbiomed.2011.09.011 21983434 10.1016/j.freeradbiomed.2011.09.011 PMC3235406

[6] Hurd TR, Requejo R, Filipovska A, Brown S, Prime TA, Robinson AJ, et al (2008) Complex I within oxidatively stressed bovine heart mitochondria is glutathionylated on Cys-531 and Cys-704 of the 75-kDa subunit: potential role of CYS residues in decreasing oxidative damage. J Biol Chem 283: 24801–24815. doi: 10.1074/jbc.M803432200 18611857 10.1074/jbc.M803432200 PMC2529008

[7] Beer SM, Taylor ER, Brown SE, Dahm CC, Costa NJ, Runswick MJ, et al (2004) Glutaredoxin 2 catalyzes the reversible oxidation and glutathionylation of mitochondrial membrane thiol proteins: implications for mitochondrial redox regulation and antioxidant DEFENSE. J Biol Chem 279: 47939–47951. doi: 10.1074/jbc.M408011200 15347644 10.1074/jbc.M408011200

[8] Mailloux RJ, Xuan JY, McBride S, Maharsy W, Thorn S, Holterman CE, et al (2014) Glutaredoxin-2 is required to control oxidative phosphorylation in cardiac muscle by mediating deglutathionylation reactions. J Biol Chem 289: 14812–14828. doi: 10.1074/jbc.M114.550574 24727547 10.1074/jbc.M114.550574 PMC4031535

[9] O’Brien M, Chalker J, Slade L, Gardiner D, Mailloux RJ (2017) Protein S-glutathionylation alters superoxide/hydrogen peroxide emission from pyruvate dehydrogenase complex. Free Radic Biol Med 106: 302–314. doi: 10.1016/j.freeradbiomed.2017.02.046 28242228 10.1016/j.freeradbiomed.2017.02.046

[10] Mailloux RJ, Craig Ayre D, Christian SL (2016) Induction of mitochondrial reactive oxygen species production by GSH mediated S-glutathionylation of 2-oxoglutarate dehydrogenase. Redox Biol 8: 285–297. doi: 10.1016/j.redox.2016.02.002 26928132 10.1016/j.redox.2016.02.002 PMC4776629

[11] Chalker J, Gardiner D, Kuksal N, Mailloux RJ (2017) Characterization of the impact of glutaredoxin-2 (GRX2) deficiency on superoxide/hydrogen peroxide release from cardiac and liver mitochondria. Redox Biol 15: 216–227. doi: 10.1016/j.redox.2017.12.006 29274570 10.1016/j.redox.2017.12.006 PMC5773472

[12] Kramer PA, Duan J, Qian WJ, Marcinek DJ (2015) The Measurement of Reversible Redox Dependent Post-translational Modifications and Their Regulation of Mitochondrial and Skeletal Muscle Function. Front Physiol 6: 347 doi: 10.3389/fphys.2015.00347 26635632 10.3389/fphys.2015.00347 PMC4658434

[13] Pastore A, Piemonte F (2012) S-Glutathionylation signaling in cell biology: progress and prospects. Eur J Pharm Sci 46: 279–292. doi: 10.1016/j.ejps.2012.03.010 22484331 10.1016/j.ejps.2012.03.010

[14] Pastore A, Piemonte F (2013) Protein glutathionylation in cardiovascular diseases. Int J Mol Sci 14: 20845–20876. doi: 10.3390/ijms141020845 24141185 10.3390/ijms141020845 PMC3821647

[15] Alegre-Cebollada J, Kosuri P, Giganti D, Eckels E, Rivas-Pardo JA, Hamdani N, et al (2014) S-glutathionylation of cryptic cysteines enhances titin elasticity by blocking protein folding. Cell 156: 1235–1246. doi: 10.1016/j.cell.2014.01.056 24630725 10.1016/j.cell.2014.01.056 PMC3989842

[16] Lewis NA, Howatson G, Morton K, Hill J, Pedlar CR (2015) Alterations in redox homeostasis in the elite endurance athlete. Sports Med 45: 379–409. doi: 10.1007/s40279-014-0276-5 25319354 10.1007/s40279-014-0276-5

[17] Mailloux RJ, Seifert EL, Bouillaud F, Aguer C, Collins S, Harper ME (2011) Glutathionylation acts as a control switch for uncoupling proteins UCP2 and UCP3. J Biol Chem 286: 21865–21875. doi: 10.1074/jbc.M111.240242 21515686 10.1074/jbc.M111.240242 PMC3122241

[18] Mailloux RJ, Xuan JY, Beauchamp B, Jui L, Lou M, Harper ME (2013) Glutaredoxin-2 is required to control proton leak through uncoupling protein-3. J Biol Chem 288: 8365–8379. doi: 10.1074/jbc.M112.442905 23335511 10.1074/jbc.M112.442905 PMC3605654

[19] Anderson EJ, Lustig ME, Boyle KE, Woodlief TL, Kane DA, Lin CT, et al (2009) Mitochondrial H2O2 emission and cellular redox state link excess fat intake to insulin resistance in both rodents and humans. J Clin Invest 119: 573–581. doi: 10.1172/JCI37048 19188683 10.1172/JCI37048 PMC2648700

[20] Zhang Y, Davis C, Sakellariou GK, Shi Y, Kayani AC, Pulliam D, et al (2013) CuZnSOD gene deletion targeted to skeletal muscle leads to loss of contractile force but does not cause muscle atrophy in adult mice. FASEB J 27: 3536–3548. doi: 10.1096/fj.13-228130 23729587 10.1096/fj.13-228130 PMC3752542

[21] Powers SK, Duarte J, Kavazis AN, Talbert EE (2010) Reactive oxygen species are signalling molecules for skeletal muscle adaptation. Exp Physiol 95: 1–9. doi: 10.1113/expphysiol.2009.050526 19880534 10.1113/expphysiol.2009.050526 PMC2906150

[22] Hill BG, Higdon AN, Dranka BP, Darley-Usmar VM (2010) Regulation of vascular smooth muscle cell bioenergetic function by protein glutathiolation. Biochim Biophys Acta 1797: 285–295. doi: 10.1016/j.bbabio.2009.11.005 19925774 10.1016/j.bbabio.2009.11.005 PMC2812626

[23] Giustarini D, Galvagni F, Tesei A, Farolfi A, Zanoni M, Pignatta S, et al (2015) Glutathione, glutathione disulfide, and S-glutathionylated proteins in cell cultures. Free Radic Biol Med 89: 972–981. doi: 10.1016/j.freeradbiomed.2015.10.410 26476010 10.1016/j.freeradbiomed.2015.10.410

[24] Giangregorio N, Palmieri F, Indiveri C (2013) Glutathione controls the redox state of the mitochondrial carnitine/acylcarnitine carrier Cys residues by glutathionylation. Biochim Biophys Acta 1830: 5299–5304. doi: 10.1016/j.bbagen.2013.08.003 23948593 10.1016/j.bbagen.2013.08.003

[25] Rossi R, Giustarini D, Dalle-Donne I, Milzani A (2006) Protein S-glutathionylation and platelet anti-aggregating activity of disulfiram. Biochem Pharmacol 72: 608–615. doi: 10.1016/j.bcp.2006.05.021 16815310 10.1016/j.bcp.2006.05.021

[26] Shelton MD, Chock PB, Mieyal JJ (2005) Glutaredoxin: role in reversible protein s-glutathionylation and regulation of redox signal transduction and protein translocation. Antioxid Redox Signal 7: 348–366. doi: 10.1089/ars.2005.7.348 15706083 10.1089/ars.2005.7.348

[27] Brand MD (2016) Mitochondrial generation of superoxide and hydrogen peroxide as the source of mitochondrial redox signaling. Free Radic Biol Med 100: 14–31. doi: 10.1016/j.freeradbiomed.2016.04.001 27085844 10.1016/j.freeradbiomed.2016.04.001

[28] Mailloux RJ, Gardiner D, O’Brien M (2016) 2-Oxoglutarate dehydrogenase is a more significant source of O2(.-)/H2O2 than pyruvate dehydrogenase in cardiac and liver tissue. Free Radic Biol Med 97: 501–512. doi: 10.1016/j.freeradbiomed.2016.06.014 27394173 10.1016/j.freeradbiomed.2016.06.014

[29] Kuksal N, Chalker J, Mailloux RJ (2017) Progress in understanding the molecular oxygen paradox—function of mitochondrial reactive oxygen species in cell signaling. Biol Chem 398: 1209–1227. doi: 10.1515/hsz-2017-0160 28675747 10.1515/hsz-2017-0160

[30] Jones DP, Sies H (2015) The Redox Code. Antioxid Redox Signal 23: 734–746. doi: 10.1089/ars.2015.6247 25891126 10.1089/ars.2015.6247 PMC4580308

[31] Gallogly MM, Mieyal JJ (2007) Mechanisms of reversible protein glutathionylation in redox signaling and oxidative stress. Curr Opin Pharmacol 7: 381–391. doi: 10.1016/j.coph.2007.06.003 17662654 10.1016/j.coph.2007.06.003

[32] McLain AL, Cormier PJ, Kinter M, Szweda LI (2013) Glutathionylation of alpha-ketoglutarate dehydrogenase: the chemical nature and relative susceptibility of the cofactor lipoic acid to modification. Free Radic Biol Med 61: 161–169. doi: 10.1016/j.freeradbiomed.2013.03.020 23567190 10.1016/j.freeradbiomed.2013.03.020 PMC3883985

[33] Chen YR, Chen CL, Pfeiffer DR, Zweier JL (2007) Mitochondrial complex II in the post-ischemic heart: oxidative injury and the role of protein S-glutathionylation. J Biol Chem 282: 32640–32654. doi: 10.1074/jbc.M702294200 17848555 10.1074/jbc.M702294200

[34] Mailloux RJ, Willmore WG (2014) S-glutathionylation reactions in mitochondrial function and disease. Front Cell Dev Biol 2: 68 doi: 10.3389/fcell.2014.00068 25453035 10.3389/fcell.2014.00068 PMC4233936

[35] Perevoshchikova IV, Quinlan CL, Orr AL, Gerencser AA, Brand MD (2013) Sites of superoxide and hydrogen peroxide production during fatty acid oxidation in rat skeletal muscle mitochondria. Free Radic Biol Med 61: 298–309. doi: 10.1016/j.freeradbiomed.2013.04.006 23583329 10.1016/j.freeradbiomed.2013.04.006 PMC3871980

[36] Chouchani ET, Pell VR, Gaude E, Aksentijevic D, Sundier SY, Robb EL, et al (2014) Ischaemic accumulation of succinate controls reperfusion injury through mitochondrial ROS. Nature 515: 431–435. doi: 10.1038/nature13909 25383517 10.1038/nature13909 PMC4255242

[37] Queiroga CS, Almeida AS, Martel C, Brenner C, Alves PM, Vieira HL (2010) Glutathionylation of adenine nucleotide translocase induced by carbon monoxide prevents mitochondrial membrane permeabilization and apoptosis. J Biol Chem 285: 17077–17088. doi: 10.1074/jbc.M109.065052 20348099 10.1074/jbc.M109.065052 PMC2878049

[38] Mailloux RJ, Fu A, Robson-Doucette C, Allister EM, Wheeler MB, Screaton R, et al (2012) Glutathionylation state of uncoupling protein-2 and the control of glucose-stimulated insulin secretion. J Biol Chem 287: 39673–39685. doi: 10.1074/jbc.M112.393538 23035124 10.1074/jbc.M112.393538 PMC3501076

[39] Pfefferle A, Mailloux RJ, Adjeitey CN, Harper ME (2013) Glutathionylation of UCP2 sensitizes drug resistant leukemia cells to chemotherapeutics. Biochim Biophys Acta 1833: 80–89. doi: 10.1016/j.bbamcr.2012.10.006 23069211 10.1016/j.bbamcr.2012.10.006

[40] Chouchani ET, Kazak L, Jedrychowski MP, Lu GZ, Erickson BK, Szpyt J, et al (2016) Mitochondrial ROS regulate thermogenic energy expenditure and sulfenylation of UCP1. Nature 532: 112–116. doi: 10.1038/nature17399 27027295 10.1038/nature17399 PMC5549630

[41] Halestrap AP, Brenner C (2003) The adenine nucleotide translocase: a central component of the mitochondrial permeability transition pore and key player in cell death. Curr Med Chem 10: 1507–1525. 12871123 10.2174/0929867033457278

[42] Kunji ER (2004) The role and structure of mitochondrial carriers. FEBS Lett 564: 239–244. doi: 10.1016/S0014-5793(04)00242-X 15111103 10.1016/S0014-5793(04)00242-X

[43] Mailloux RJ, McBride SL, Harper ME (2013) Unearthing the secrets of mitochondrial ROS and glutathione in bioenergetics. Trends Biochem Sci 38: 592–602. doi: 10.1016/j.tibs.2013.09.001 24120033 10.1016/j.tibs.2013.09.001

